# Case Report: Opportunities and Challenges of Immunotherapy in Heavily-Treated EGFR-Mutant Advanced Squamous Cell Lung Carcinoma After Progression on EGFR-TKIs and Chemotherapy

**DOI:** 10.3389/fonc.2022.820408

**Published:** 2022-02-28

**Authors:** Wei Jin, Xin Wang, Jie Wang, Lin Lin

**Affiliations:** ^1^ Department of Chinese Medicine, National Cancer Center/National Clinical Research Center for Cancer/Cancer Hospital, Chinese Academy of Medical Sciences and Peking Union Medical College, Beijing, China; ^2^ Department of Pathology, National Cancer Center/National Clinical Research Center for Cancer/Cancer Hospital, Chinese Academy of Medical Sciences and Peking Union Medical College, Beijing, China; ^3^ Department of Medical Oncology, National Cancer Center/National Clinical Research Center for Cancer/Cancer Hospital, Chinese Academy of Medical Sciences and Peking Union Medical College, Beijing, China

**Keywords:** immunotherapy, camrelizumab, epidermal growth factor receptor (EGFR) mutations, advanced squamous cell lung carcinoma (SqCLC), case report

## Abstract

**Background:**

Epidermal growth factor receptor (EGFR) mutations have a low incidence in squamous cell lung cancer (SqCLC), and the clinical efficacy of EGFR tyrosine kinase inhibitors (TKIs) in *EGFR*-mutated SqCLC is far less than that in *EGFR*-mutated lung adenocarcinoma. The treatment strategy for patients with *EGFR-*mutated non-small cell lung cancer who are refractory to EGFR TKIs has become a current dilemma and challenge.

**Case Presentation:**

A case of a 69-year-old male patient suffering from intermittent cough and hemoptysis was diagnosed with *EGFR*-mutated advanced SqCLC (stage cT2bN2M1). The patient was treated with camrelizumab alone after five courses of different systemic therapies and achieved a partial response, with an eminent progression-free survival of more than 24 months. Grade 1 to 2 reactive cutaneous capillary endothelial proliferation and mild pruritus were observed during the treatment. No other immune-related adverse events were observed.

**Conclusion:**

Monotherapy of immune-checkpoint inhibitors may be considered as a later-line option for *EGFR*-mutated advanced SqCLC patients with PD-L1 expression.

## Introduction

Lung cancer is currently the malignant tumor with the highest morbidity and mortality around the world. Squamous cell lung carcinoma (SqCLC) is a common pathological type of lung cancer, accounting for about 25 to 30% in non-small cell lung cancer (NSCLC) (1, 2). Epidermal growth factor receptor (EGFR) mutation is one of the most common driver mutations in NSCLC but occur in only 3.3–4.6% of patients with SqCLC (3). Patients with *EGFR*-mutated advanced SqCLC benefit limitedly from EGFR tyrosine kinase inhibitors (TKIs). In 2013, Fang *et al.* reported that the objective response rate (ORR) and median progression-free survival (mPFS) of first-generation EGFR-TKIs (gefitinib or erlotinib) in 15 advanced SqCLC patients with *EGFR* mutation was 26.7% and 3.9 months, respectively (4). Another pooled analysis from Japan demonstrated that the mPFS of gefitinib in 27 *EGFR*-mutated SqCLC patients was only 3.1 months, with an ORR of 30% (5). As the resistance to targeted therapy is inevitable and the mechanisms are complex, the treatment strategy after EGFR-TKI resistance has become a dilemma and challenge for patients with *EGFR*-mutated advanced NSCLC. With the advent of immunotherapy, a variety of immune-checkpoint inhibitors (ICIs) targeting programmed cell death protein 1/programmed cell death ligand 1 (PD-1/PD-L1) have been proven to benefit patients with advanced NSCLC (6–10). However, only about 20% of NSCLC patients could benefit from immunotherapy in second-line treatment (8). Due to the complex clinical features and gene mutations of SqCLC, which make its treatment challenging, whether patients with *EGFR*-mutated advanced NSCLC can benefit from immunotherapy has become a hot issue.

Here we describe a case of *EGFR*-mutated advanced SqCLC treated with PD-1 inhibitor monotherapy and which achieved an eminent PFS of 24.4 months in the sixth-line treatment and provide an assessment of the efficacy and safety of the treatment.

## Background

**Figure 1:**
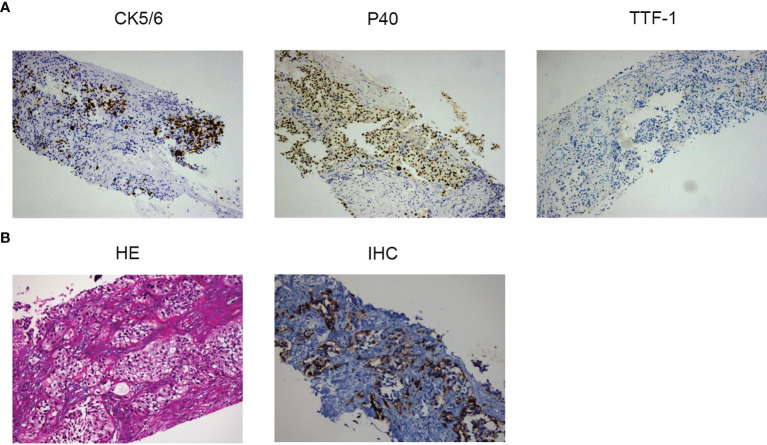
**(A)** Histopathology of the left upper lobe mass at diagnosis: expression of cell keratin 5/6 and P40 thyroid transcription factor-1 by immunohistochemistry (original magnification, ×10). **(B)** Pathologic findings of the patient’s right kidney metastasis before the sixth-line treatment with camrelizumab: hematoxylin and eosin staining of PD-L1 (original magnification, ×20) and immunohistochemistry of PD-L1 (original magnification, ×10).

In June 2018, a 69-year-old male with a 15-year smoking history went to our hospital; he had been suffering from intermittent cough and hemoptysis for 3 months. An enhanced computed tomography (CT) scan of the chest revealed a proximal interlobular pleural mass in the left lung upper lobe, multiple nodules and masses in the left lung lower lobe, a mass-like opacity in the right lung upper lobe, and a right renal mass. SqCLC was confirmed by pathological examination and immunohistochemistry study of CT-guided biopsy specimens from the mass of the left upper lobe (**Figure 1A**). The patient’s condition was staged as cT2bN2M1 (stage IV) according to the 8th Edition of the American Joint Committee on Cancer TNM Staging System (2017). The next-generation sequencing (NGS) analysis of the patient’s peripheral blood sample with a panel (Repugene Technology, Hangzhou, China) including 18 somatic genes related to NSCLC and the efficacy of targeted drugs suggested *EGFR Exon 21 L858R*, *G719A*, and *S768I* mutations. Other driver gene mutations, such as *ALK*, *ROS1*, *ERBB2* (*HER2*), *BRAF*, *KRAS*, *TP53*, *PIK3CA*, *etc.*, were not detected.

**Figure 2:**
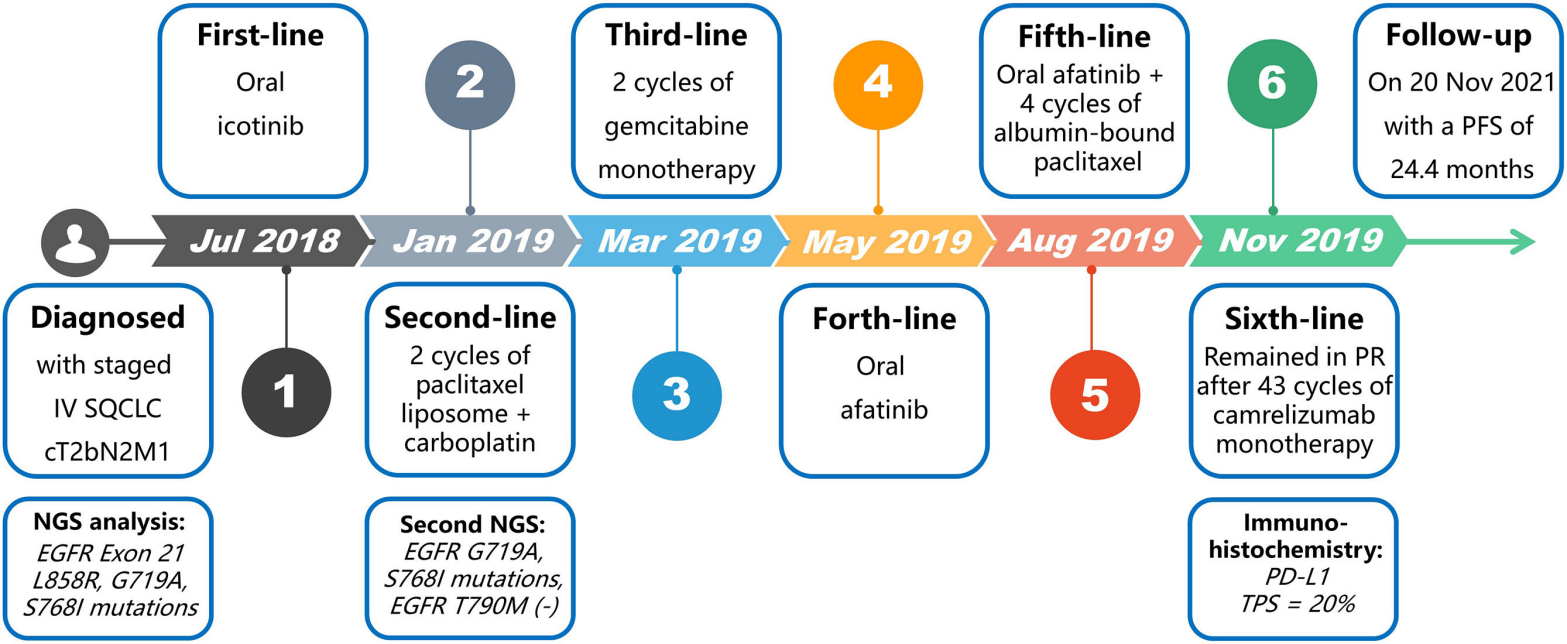
Diagnosis and treatment procedure.

In July 2018, with an Eastern Cooperative Oncology Group performance status score of 0, the patient received first-line treatment with icotinib (**Figure 2**), a first-generation EGFR-TKI, and achieved a partial response (PR). A CT scan in January 2019 revealed multiple nodules in the right lung and enlarged lesions in the left lung and right kidney, indicating a progressive disease (PD). The PFS for the first-line treatment was 6 months.

The second NGS analysis of the whole blood sample with a large panel (Genetron Health, Beijing, China), including 824 somatic genes, suggested *EGFR G719A* and *S768I* mutations and negative *EGFR* T790M mutation. On January 24, 2019, the pathological examination of an ultrasound-guided biopsy of the right renal mass revealed a poorly differentiated squamous cell carcinoma, which may be derived from tumor metastasis. On January 30, 2019, the second-line treatment consisting of paclitaxel liposome in combination with carboplatin was administered. A re-examination after 2 cycles of treatment revealed enlarged primary and metastatic lesions, along with lymph node metastases presenting between the abdominal aorta and inferior vena cava, suggesting PD. The second-line therapy was continued for 1.2 months.

The third-line treatment with 2 cycles of gemcitabine monotherapy was started on March 17, 2019 and was continued for 2.2 months. Then, the disease progressed with bilateral frontal lobe metastases as revealed by magnetic resonance imaging.

The treatment was switched to oral afatinib, a second-generation *EGFR*-TKI, in May 2019 and lasted 2.8 months until the disease progressed with new liver metastases.

**Figure 3:**
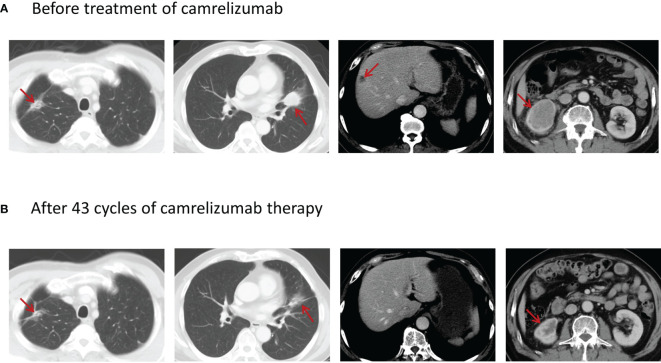
**(A)** Enhanced CT scan before the treatment of camrelizumab showing a mass-like opacity in the upper lobe of the right lung with a maximum cross-sectional area of about 3.4 × 1.4 cm, a 3.3 × 2.8-cm tumor in the upper lobe of the left lung near the interlobar pleura, a low-density subcapsular nodule in the right lobe of the liver of about 1.5 × 1.4 cm in size, and a metastatic tumor in the lower right kidney with the size of about 5.9 × 4.3 cm. **(B)** Enhanced CT scan after 43 cycles of treatment with camrelizumab showing that the tumor in the upper lobe of the right lung was of 3.1 × 2.3 cm in maximum cross-sectional area, the tumor in the upper lobe of the left lung near the interlobar pleura decreased to the size of 1.7 × 0.8 cm, the subcapsular lesion in the right lobe of the liver was calcified, and the tumor in the lower right kidney decreased to the size of 2.6 × 1.9 cm.

On August 26, 2019, the patient was given a systemic chemotherapy of afatinib combined with albumin-bound paclitaxel. Nevertheless, his disease progressed again after 4 cycles of this fifth-line treatment, as the re-examination suggested enlargement of the liver metastases and the right kidney metastasis (**Figure 3A**), with a PFS of 2.6 months.

Since an immunohistochemistry study of the right kidney metastasis showed a PD-L1 tumor proportion score (TPS) of 20% (22c3 antibody on the Dako Link-48 platform) (**Figure 1B**), single-agent camrelizumab (200 mg every 2 weeks) was started on November 19, 2019. The patient’s cough was obviously relieved, and the re-examination indicated a stable disease after 3 cycles of camrelizumab. PR was achieved after 6 cycles and confirmed again by the response evaluation after 10 cycles. The patient remained in PR after 43 cycles (**Figure 3B**) and was about to receive the 50th cycle of monotherapy with camrelizumab as of the time of writing of this case report (November 20, 2021), with a PFS of 24.4 months.

**Figure 4:**
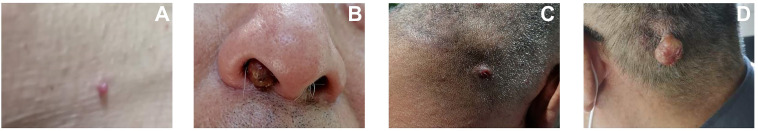
Reactive cutaneous capillary endothelial proliferation. **(A)** “Red-nevus-like” nodule with a diameter of 1 to 2 mm on the skin surface of the neck. **(B)** “Tumor-like” nodule with a diameter of >10 mm in the nasal cavity. **(C)** “Pearl-like” nodule with a diameter of 5 mm on the occipital skin surface. **(D)** The nodule on the left occipital skin surface enlarged to a pedicled “tumor-like” nodule with a diameter of >10 mm.

After 6 cycles of camrelizumab, the patient presented with reactive cutaneous capillary endothelial proliferation (RCCEP), multiple bright red spots (“red-nevus-like”), with a spot on the skin surface of the neck measuring 1 to 2 mm in diameter (grade 1 RCCEP) (**Figure 4A**) and with a “pearl-like” nodule in the nasal cavity. After 8 cycles of camrelizumab, the nodule in the nasal cavity developed into a “tumor-like” nodule, >10 mm in diameter (**Figure 4B**), along with several “pearl-like” nodules on the occipital skin surface at 5 mm in diameter (**Figure 4C**). The nodule on the left occipital skin surface enlarged to a pedicled “tumor-like” nodule, >10 mm in diameter (**Figure 4D**), after 10 cycles of camrelizumab. Both “tumor-like” and “pearl-like” nodules on the surface of the nasal cavity and the occiput were treated with laser and underwent resection. After 35 cycles of camrelizumab, the patient developed pruritus all over the body and the extremities (grade 1 under the Common Terminology Criteria for Adverse Events). During the sixth-line treatment with camrelizumab, there was no ulceration, hemorrhage (grade 2 RCCEP), or other immune-related adverse events (irAEs) observed except for RCCEP.

## Discussion

*EGFR* mutation is an important molecular marker of NSCLC. *EGFR Exon 21 L858R* mutation, a classical activating mutation, is considered related to a better outcome in NSCLC patients, granting a complete blockade of the EGFR signaling pathway by EGFR-TKIs (11). The 6 months of PFS of the first-line treatment with icotinib and the *L858R* mutation that disappeared in the second NGS testing after icotinib treatment in our case demonstrated the *L858R* mutation as a positive predictor of clinical prognosis which responds well to first-generation EGFR-TKI. *G719A* mutation is one of the common *EGFR exon 18* mutations, which was reported as a predictor of augmented sensitivity to afatinib or neratinib (12). The *EGFR exon 20 S768I* mutation is a rare mutation at a frequency of between 0.6 and 1% of EGFR mutations in NSCLC (13) and often co-occurs with other *EGFR* point mutations to form complex mutations—for example, S*768I/G719X* (*G719X* including *G719S*, *G719A*, *G719C*, and *G719D* substitutions) or *S768I/L858R* complex mutations. FDA approved afatinib for NSCLC patients with *EGFR S768I* mutations based on 100% of ORR and 14.7 months of mPFS for eight patients with *S768I* mutant NSCLC in a *post-hoc* analysis (14). Furthermore, the *S768I/G719A* co-mutation was confirmed to be resistant to first-generation and highly sensitive to afatinib (15). Thus, in our case, we used afatinib for fourth-line treatment in the SqCLC patient with a *S768I/G719A* co-mutation. However, unfortunately, gene detections after progression in the later-line treatments were lacking due to realistic reasons, and whether the genetic mutation changed during the different treatments is unknown, so the mechanism of repeated resistance to EGFR-TKIs and chemotherapies is uncertain.

EGFR-TKI is a standard treatment option for *EGFR*-mutated NSCLC, but the treatment strategy after drug resistance to EGFR-TKIs has been a great challenge. There is an urgent need for immunotherapy to achieve the precise treatment of *EGFR*-mutated NSCLC patients, bringing new hope to *EGFR*-mutated NSCLC patients. However, patients with *EGFR* mutations were once considered to be “immune-privilege” populations. A subgroup analysis of most previous studies showed little benefit from mono-immunotherapy in *EGFR/ALK*-mutated patients. A phase II clinical trial (NCT02879994) explored ICI monotherapy as the first-line treatment for *EGFR*-mutated NSCLC with 11 patients enrolled, but the recruitment was stopped due to lack of efficacy. Most clinical trials of first-line mono-immunotherapy excluded patients with *EGFR*-mutated NSCLC.

Regarding the second-line treatment with ICIs, the outcomes of the CHECKMATE-057 study (8) showed that the PFS and the overall survival (OS) were not significantly improved with nivolumab than with docetaxel among patients with *EGFR*-mutated advanced non-squamous NSCLC that had progressed after chemotherapy treatment. The KEYNOTE-010 study (pembrolizumab) (16) and the OAK study (atezolizumab) (10) showed similar results that revealed no OS benefit in *EGFR*-mutated subgroups. A retrospective study from Massachusetts General Hospital, which enrolled 28 patients with *EGFR+/ALK+* NSCLC, indicated a significantly lower ORR of immunotherapy in *EGFR+/ALK+* patients than that of *EGFR-/ALK-* patients (3.6 *vs*. 23.3%) (17). Other meta-analyses, including CHECKMATE-057, KEYNOTE-010, and POPLAR studies, showed that patients with *EGFR*-mutated advanced NSCLC cannot benefit from ICIs (18).

Although many studies have confirmed that *EGFR*-mutated advanced NSCLC is not sensitive to immunotherapy, two patients in the CA209-003 study had progressed after more than two lines of treatment, received nivolumab monotherapy, and survived for more than 5 years in the follow-up (19). In 2020, the ATLANTIC study (20), the largest prospective trial of anti-PD-1/PD-L1 treatments for patients with positive driver gene mutations so far, updated its results. In this trial, eligible patients were assigned into three groups based on the status of *EGFR/ALK* mutations and PD-L1 expression to evaluate the safety and efficacy of durvalumab for third-line or above treatment and found that the ORR (12.2 *vs*. 3.6%) and mOS (13.3 *vs*. 10.9 months) of patients with *EGFR+/ALK+* NSCLC with a PD-L1 TPS of **≥**25% were higher than those with *EGFR-/ALK-* NSCLC with a PD-L1 TPS of **≥**25%. These results denied the previous conclusion that *EGFR+/ALK+* patients are not fit for immunotherapy. For patients with *EGFR* mutations, PD-L1 may be used as a biomarker to predict the clinical efficacy of immunotherapy, which may provide another option of the third- or later-line treatment for *EGFR*-mutated NSCLC with PD-L1 overexpression. However, most of the patients included in the above-mentioned studies were *EGFR*-mutated lung adenocarcinoma case. In the *EGFR+/ALK+* group of the ATLANTIC study, only one of 111 patients was a case of SqCLC. There was no detailed report on this rare case.

Immunotherapy for EGFR-mutant patients is faced with opportunities and challenges. Combination immunotherapy is becoming a new exploration direction. Pre-clinical studies found that EGFR activation upregulated PD-L1 through p-ERK1/2/p-c-Jun and induced the apoptosis of T cells. Accordingly, EGFR-TKIs could alleviate the inhibition effect of PD-L1/PD-1 axis on T cells and increase the production of interferon-γ. Moreover, PD-L1 expression is significantly elevated in EGFR-TKI-resistant cell lines, whereas anti-PD-1 antibody could suppress the viability of EGFR-TKI-resistant NSCLC cells (21), which may explain the good response to camrelizumab after repeated drug resistance to EGFR-TKIs in our case. Considering the potential interaction of PD-L1/PD-1 inhibitors and EGFR-TKIs, a number of trials regarding the combination therapy of ICIs with EGFR-TKIs have been conducted, such as the cohort of nivolumab combined with erlotinib in CheckMate 012 study (22), the KEYNOTE-021 study of pembrolizumab in combination with erlotinib or gefitinib (23), the phase-I open-label, multicenter trial of durvalumab plus gefitinib (NCT02088112) (24), the TATTON study of durvalumab combined with osimertinib (25), and the phase Ib study of atezolizumab plus erlotinib (26). The results of these studies suggest that ICIs combined with EGFR-TKIs can achieve various degrees of ORR but will also cause intolerable toxicity at the same time. In particular, due to the high incidence of interstitial pneumonitis during the treatment of durvalumab plus osimertinib, the TATTON study and the phase III CAURAL study were terminated. In addition, the study of CTLA-4 inhibitors plus targeted therapy suspended the recruitment (27) due to dose-limiting toxicity. Currently, the development of immune-based combinations remains a crucial unmet need. To explore the optimal combination strategy of immune-based therapy, reducing its toxic side effects and revealing its efficacy mechanism in *EGFR*-mutated NSCLC patients will be among the challenges for future research in this field.

ICIs combined with chemotherapy and anti-angiogenic drugs is another combination treatment strategy. The IMpower150 study is a phase III clinical study of the first-line treatment of advanced non-squamous NSCLC (28), and the subgroup analysis showed a significant improvement in OS (median OS: 29.4 *vs*. 18.1 months, HR = 0.60; 95% CI: 0.31–1.14) of ICIs combined with chemotherapy and anti-angiogenic therapy (atezolizumab + bevacizumab + carboplatin + paclitaxel) compared with that of chemotherapy combined with anti-angiogenic therapy (bevacizumab + carboplatin + paclitaxel) among 124 *EGFR*-mutated non-squamous NSCLC patients who had a failed previous targeted therapy. The IMpower150 study is currently the only phase III clinical trial in which immune combination therapy brought a survival benefit to *EGFR*-mutated patients. This global multi-center study overturns the theory of immune privilege resulting from *EGFR* mutations and provides new opportunities for the later-line treatment after EGFR-TKI resistance.

Camrelizumab is an IgG4-humanized monoclonal antibody against PD-1, which can inhibit the binding of PD-L1 and PD-L2 to PD-1, and has a promising anti-tumor efficacy. The patient with *EGFR*-mutated SqCLC in this paper has received multiple courses of targeted therapy, chemotherapy, and the combination of chemotherapy with targeted therapy, but the disease has progressed rapidly. Based on the PD-L1 expression status of the metastasis in the right kidney, the patient received 49 cycles of mono-immunotherapy with camrelizumab and achieved PR. The patient is still receiving maintenance treatment with camrelizumab alone, with a PFS of more than 24 months. This case report provides further evidence that a small number of patients with *EGFR*-mutated advanced SqCLC can benefit from mono-immunotherapy. Thus, mono-immunotherapy still has the opportunity to play an important role in the later-line treatment of *EGFR*-mutated advanced SqCLC, especially after multiple courses of different types of therapy, and PD-L1 may be used as a biomarker to predict the clinical efficacy of immunotherapy. The mechanism related to the prominent response to camrelizumab monotherapy and whether *S768I/G719A* mutation is one of the possible mechanisms need more inspection and exploration.

As with clinical trials, case reports regarding the treatment of ICIs for *EGFR*-mutated NSCLC are mostly cases of lung adenocarcinoma (29–31) or about a changed phenotype from lung adenocarcinoma to SqCLC (32). The patient in this case was first diagnosed with SqCLC and has a longer PFS (>24 *vs*. 16 months) during the treatment with camrelizumab compared with that of the patient with *EGFR*-mutated NSCLC in the CA209-003 trial who received durvalumab as a third-line treatment and had an OS of >5 years (19).

The patient developed grade 1 to 2 RCCEP during the treatment of camrelizumab, without other irAEs. RCCEP, a common, typical irAE of camrelizumab, mainly occurred on the skin surface of the head, neck, trunk, and extremities but rarely in other parts such as the oral mucosa, nasal mucosa, and eyelid conjunctiva and was never observed in visceral organs (33, 34). The mechanism of RCCEP is unclear and may be related to the imbalance between angiogenesis accelerants and inhibitors. Most RCCEPs are self-limited and have little impact on the quality of life. However, for patients with a high risk of bleeding, if the RCCEP is severely congested, fragile, located in the friction sites, or repeatedly bleeding, local resection may be a better choice. RCCEP may be used as a marker to predict the efficacy of camrelizumab. Studies have found that patients who develop RCCEP during the treatment with camrelizumab have an ORR of 28.9%, while patients without RCCEP do not respond to camrelizumab (33). In this report, the patient developed “red-nevus-like”, “pearl-like”, and “tumor-like” nodules, and both “pearl-like” and “tumor-like” nodules had been removed. Although the patient had grade 1 to 2 RCCEP, the immune response was obvious, consistent with the results previously reported in the literatures.

## Concluding Remarks

At present, EGFR-TKIs are still the standard regimen for *EGFR*-mutated advanced NSCLC, but due to the complex drug resistance mechanism, mono-immunotherapy or a combination therapy of immunotherapy with other treatments needs to be optimized. The studies mentioned before have shown that the vast majority of patients with advanced NSCLC refractory to EGFR-TKIs had a poor curative effect to ICIs. However, in view of the fact that ICIs could exert anti-tumor effects through the patients’ own immune system, there are still a small number of *EGFR*-mutated advanced NSCLC that can benefit from immunotherapy after drug resistance to EGFR-TKIs. Mono-immunotherapy could be considered as one of the later-line treatment options for patients with *EGFR*-mutated advanced SqCLC, especially for those without *EGFR* T790M mutation or with high PD-L1 expression. The national, multi-center, randomized, double-blind phase III TREASURE study, CHECKMATE-722 study, KEYNOTE-789 study, and ORIENT-31 study are ongoing to explore the efficacy of immunotherapy for patients with NSCLC after PD on EGFR-TKIs. More and more clinical and basic research concerns were expected in these patients to further screen the advantageous population of ICIs in the treatment of *EGFR*-mutated advanced SqCLC, to find predictive biomarkers of clinical efficacy, and to achieve a personalized precision treatment.

## Data Availability Statement

The original contributions presented in the study are included in the article/supplementary material. Further inquiries can be directed to the corresponding authors.

## Ethics Statement

The studies involving human participants were reviewed and approved by the Ethics Committee of the National Cancer Center/National Clinical Research Center for Cancer/Cancer Hospital, Chinese Academy of Medical Sciences. The patients/participants provided their written informed consent to participate in this study. Written informed consent was obtained from the individual(s) for the publication of any potentially identifiable images or data included in this article.

## Author Contributions

All authors were involved in the clinical management of the presented case and participated in manuscript preparation. All authors contributed to the article and approved the submitted version.

## Conflict of Interest

The authors declare that the research was conducted in the absence of any commercial or financial relationships that could be construed as a potential conflict of interest.

## Publisher’s Note

All claims expressed in this article are solely those of the authors and do not necessarily represent those of their affiliated organizations, or those of the publisher, the editors and the reviewers. Any product that may be evaluated in this article, or claim that may be made by its manufacturer, is not guaranteed or endorsed by the publisher.
